# Comparative effectiveness of ChAdOx1 versus BNT162b2 covid-19 vaccines in health and social care workers in England: cohort study using OpenSAFELY

**DOI:** 10.1136/bmj-2021-068946

**Published:** 2022-07-20

**Authors:** William J Hulme, Elizabeth J Williamson, Amelia C A Green, Krishnan Bhaskaran, Helen I McDonald, Christopher T Rentsch, Anna Schultze, John Tazare, Helen J Curtis, Alex J Walker, Laurie A Tomlinson, Tom Palmer, Elsie M F Horne, Brian MacKenna, Caroline E Morton, Amir Mehrkar, Jessica Morley, Louis Fisher, Sebastian C J Bacon, David Evans, Peter Inglesby, George Hickman, Simon Davy, Tom Ward, Richard Croker, Rosalind M Eggo, Angel Y S Wong, Rohini Mathur, Kevin Wing, Harriet Forbes, Daniel J Grint, Ian J Douglas, Stephen J W Evans, Liam Smeeth, Chris Bates, Jonathan Cockburn, John Parry, Frank Hester, Sam Harper, Jonathan A C Sterne, Miguel A Hernán, Ben Goldacre

**Affiliations:** 1 The Bennett Institute for Applied Data Science, Nuffield Department of Primary Care Health Sciences, University of Oxford, Oxford OX2 6GG, UK; 2 London School of Hygiene and Tropical Medicine, London WC1E 7HT, UK; 3 MRC Integrative Epidemiology Unit, Bristol Medical School, University of Bristol, Bristol, UK; 4 Population Health Sciences, University of Bristol, Bristol BS8 2BN, UK; 5 NIHR Bristol, Biomedical Research Centre, Bristol BS8 2BN, UK; 6 TPP, TPP House, Horsforth, Leeds LS18 5PX, UK; 7 Health Data Research UK South West; 8 CAUSALab, Harvard T.H. Chan School of Public Health, Boston, MA 02115, USA; 9 Departments of Epidemiology and Biostatistics, Harvard T.H. Chan School of Public Health, Boston, MA 02115, USA; Correspondence to: B Goldacre ben.goldacre@phc.ox.ac.uk

## Abstract

**Objective:**

To compare the effectiveness of the BNT162b2 mRNA (Pfizer-BioNTech) and the ChAdOx1 (Oxford-AstraZeneca) covid-19 vaccines against infection and covid-19 disease in health and social care workers.

**Design:**

Cohort study, emulating a comparative effectiveness trial, on behalf of NHS England.

**Setting:**

Linked primary care, hospital, and covid-19 surveillance records available within the OpenSAFELY-TPP research platform, covering a period when the SARS-CoV-2 Alpha variant was dominant.

**Participants:**

317 341 health and social care workers vaccinated between 4 January and 28 February 2021, registered with a general practice using the TPP SystmOne clinical information system in England, and not clinically extremely vulnerable.

**Interventions:**

Vaccination with either BNT162b2 or ChAdOx1 administered as part of the national covid-19 vaccine roll-out.

**Main outcome measures:**

Recorded SARS-CoV-2 positive test, or covid-19 related attendance at an accident and emergency (A&E) department or hospital admission occurring within 20 weeks of receipt of the first vaccine dose.

**Results:**

Over the duration of 118 771 person-years of follow-up there were 6962 positive SARS-CoV-2 tests, 282 covid-19 related A&E attendances, and 166 covid-19 related hospital admissions. The cumulative incidence of each outcome was similar for both vaccines during the first 20 weeks after vaccination. The cumulative incidence of recorded SARS-CoV-2 infection 20 weeks after first-dose vaccination with BNT162b2 was 21.7 per 1000 people (95% confidence interval 20.9 to 22.4) and with ChAdOx1 was 23.7 (21.8 to 25.6), representing a difference of 2.04 per 1000 people (0.04 to 4.04). The difference in the cumulative incidence per 1000 people of covid-19 related A&E attendance at 20 weeks was 0.06 per 1000 people (95% CI −0.31 to 0.43). For covid-19 related hospital admission, this difference was 0.11 per 1000 people (−0.22 to 0.44).

**Conclusions:**

In this cohort of healthcare workers where we would not anticipate vaccine type to be related to health status, we found no substantial differences in the incidence of SARS-CoV-2 infection or covid-19 disease up to 20 weeks after vaccination. Incidence dropped sharply at 3-4 weeks after vaccination, and there were few covid-19 related hospital attendance and admission events after this period. This is in line with expected onset of vaccine induced immunity and suggests strong protection against Alpha variant covid-19 disease for both vaccines in this relatively young and healthy population of healthcare workers.

## Introduction

The covid-19 global pandemic has prompted the rapid development and delivery of vaccines to combat the disease. Following demonstration of high safety and efficacy against symptomatic and severe disease in phase III randomised controlled trials (RCTs), two vaccines have been approved and widely administered as part of the national vaccination programme in the United Kingdom: the Pfizer-BioNTech BNT162b2 mRNA covid-19 vaccine (BNT162b2),^1^ and the Oxford-AstraZeneca ChAdOx1 nCoV-19 viral vector vaccine (ChAdOx1).^2^ Post-authorisation assessment of vaccine effectiveness using observational data are necessary to monitor the success of such programmes as, invariably, target populations and settings differ substantially from those of trials. To date, there have been no RCTs that have directly compared the BNT162b2 and ChAdOx1 vaccines to estimate their relative efficacy against covid-19 infection and disease in the same population. The concurrent roll-out of these vaccines across the UK,^3^ combined with the country’s well developed infrastructure for conducting research using routinely collected primary care health data, provides a rare opportunity to emulate such a trial using observational data.

Covid-19 vaccination in the UK has been prioritised based on the risk of infection and subsequent severity of disease.^4^ Health and social care workers were among the first groups eligible for vaccination due to the potentially high occupational exposure to the SARS-CoV-2 virus, making health and social care worker status an important confounder of the effect of vaccination on infection and post-infection disease. By studying this group in isolation, this confounding is mitigated. Further, unlike many other young people vaccinated later, health and social care workers were vaccinated during a period of high community infection, increasing event rates and therefore statistical power.

This cohort study used a target-trial design to assess the effectiveness of ChAdOx1 compared with BNT162b2 against covid-19-related outcomes in health and social care workers, including second dose effectiveness, using the OpenSAFELY-TPP linked primary care database covering around 40% of England’s population. Effectiveness was assessed primarily in an era where the SARS-CoV-2 Alpha variant was dominant.

## Methods

### Data source

The OpenSAFELY-TPP database (https://opensafely.org) covers 24 million people registered at National Health Service (NHS) general practices using TPP SystmOne electronic health record software. It includes pseudonymised data such as coded diagnoses, medications, and physiological parameters. No free text data are included. The primary care data are linked, via NHS numbers, with records of attendance at accident and emergency (A&E) departments and inpatient hospital admission via NHS Digital’s Hospital Episode Statistics (HES), national coronavirus testing records via the Second Generation Surveillance System (SGSS), and national death registry records from the Office for National Statistics (ONS). Vaccination status is available in the GP record directly via the National Immunisation Management System (NIMS). Self designated health and social care worker status is recorded for all vaccine recipients at the time of vaccination, and this information is sent to OpenSAFELY-TPP from NHS Digital’s covid-19 data store.

### Study population

We studied health and social care workers in England vaccinated with either BNT162b2 or ChAdOx1. This group was prioritised for vaccination at the start of the vaccine roll-out due to the potentially high occupational exposure to the SARS-CoV-2 virus, and many were vaccinated during the period where both vaccines were widely used.

Vaccinated health and social care workers were included in the study if: they were registered at a GP practice using TPP’s SystmOne clinical information system on the day that they received their first dose of BNT162b2 or ChAdOx1; the date of vaccination was between 4 January (when ChAdOx1 was first administered in England) and 28 February 2021, a 56 day period when both vaccines were administered widely; they were aged between 18 and 64 years inclusive; not classed as “clinically extremely vulnerable” in government guidance^4^ at the time of vaccination; information on sex, ethnicity, deprivation, and geographical region was known.

Study participants were followed up for no more than 20 weeks from the day of the first dose, including time after their second dose. Follow-up was censored earlier than this at 13 June 2021, death, or de-registration.

### Outcomes

Three outcomes were defined: positive SARS-CoV-2 test; covid-19 related A&E attendance; and unplanned covid-19 related hospital admission. Positive SARS-CoV-2 tests were identified using SGSS records and based on swab date. Both polymerase chain reaction (PCR) and lateral flow positive tests were included, without differentiation between symptomatic and asymptomatic infection. PCR and lateral flow tests were freely available in England during the study period for people with symptoms or recent contacts of those who tested positive. Tests were prioritised for health and social care workers and other key workers. Test provenance (for example, patient-initiated tests versus employee screening) was not known. Covid-19 related A&E attendances were identified using HES emergency care records with U07.1 (“covid-19, virus identified”) or U07.2 (“covid-19, virus not identified”) ICD-10 diagnosis codes.^5^ Unplanned covid-19 related hospital admissions were identified using HES inpatient hospital records with U07.1 or U07.2 reason for admission ICD-10 codes.

Although severe disease (such as requirement for intensive or critical care) and mortality were of interest, there were too few events to investigate these outcomes fully. Unadjusted incidence of covid-19 deaths are reported descriptively. These were identified using linked death registration data. Deaths with covid-19 ICD-10 codes (as above) mentioned anywhere on the death certificate (that is, as an underlying or contributing cause of death) were included.

### Additional variables

Participant characteristics used to describe the cohort and for confounder adjustment include age, sex (male or female), English Index of Multiple Deprivation (IMD, grouped by quintiles), ethnicity (Black, Mixed, South Asian, White, Other, as per the UK census), NHS region (East of England, Midlands, London, North East and Yorkshire, North West, South East, South West), number of conditions in the clinically “at risk” (but not clinically extremely vulnerable) classification, as per national prioritisation guidelines, the number of SARS-CoV-2 tests (positive or negative) in the 90 days before the study start date (via SGSS), rurality (urban conurbation, urban city or town, rural town or village), evidence of prior SARS-CoV-2 infection (positive test or covid-19 related hospitalisation), learning disabilities, and severe mental illness. All characteristics were ascertained as at the time of vaccination.

### Statistical analysis

We compared the effectiveness of a first dose of ChAdOx1 versus BNT162b2 using pooled logistic regression (PLR)^6^ ^7^ with time since vaccination as the timescale and with the outcome risk estimated each day. The effect is permitted to vary over the timescale to account for the potential time-varying differences in vaccine protection between the two brands. A PLR model can be used to approximate Cox models with time-varying treatment effects and enables the estimation of risk-adjusted cumulative incidence for each vaccine type. This is the average over all participants of the cumulative incidence for each day of follow-up predicted by the PLR model, under the (counterfactual) assumption that everyone received the BNT162b2 vaccine or that everyone received the ChAdOx1 vaccine. Assuming adequate confounder adjustment, this estimates the vaccine-specific cumulative incidence that would have been observed in an RCT comparing the two vaccines in the population under consideration. Standard errors for the PLR model were obtained using the clustered sandwich estimator to account for within-participant clustering. Confidence intervals for the vaccine-specific marginal cumulative incidence, and their difference, were obtained using the delta method (that is, a first-order Taylor series approximation of the variance).

The PLR model included vaccine type, and a vaccine-specific three-knot restricted cubic spline for time since vaccination. Knot locations were based on quartiles of the event times. Three models were fit for each outcome, with progressive adjustment for confounders^1^: adjusting for region-specific calendar-time effects, by including a two-knot restricted cubic spline for the date of vaccination and its interaction with region^2^; additionally adjusting for demographic characteristics^3^; additionally adjusting for clinical characteristics.

During the study period, the advice from the UK chief medical officers was that “second doses of both vaccines will be administered towards the end of the recommended vaccine dosing schedule of 12 weeks.”^8^ Using an intention-to-treat approach, comparative effectiveness estimates beyond 14 weeks were considered to be second-dose effects. We report the actual timing of second doses to assess the extent of any deviation from this recommended treatment strategy, frequency of any cross-brand second doses, and how these may differ between vaccine types.

PLR models are computationally expensive to fit, as the input dataset must be arranged as one row per person per day of follow-up. To manage this, a sampling strategy was used such that all those who experienced the event of interest are selected (to retain statistical power), and a random sample of 50 000 event-free participants are selected. Person-time is weighted by the inverse of the sampling probability to recover the characteristics of the complete cohort.

### Missing data

A complete-case approach was used to deal with missing values. After exclusions for missing values on demographic variables (see exclusion criteria), there were no missing values in remaining variables as they were defined by the presence or absence of clinical codes or events.

### Sensitivity analyses

We conducted a post-hoc sensitivity analysis that repeated the main analysis in the subgroup of participants who had no evidence of a prior SARS-CoV-2 infection. We also use piecewise-linear time-varying hazards (at 7-day intervals in the first 28 days, and 14-day intervals thereafter) to investigate whether the estimates were robust to our choice of spline parameters.

### Software, code, and reproducibility

Data management and analyses were conducted in Python 3.8 and R version 4.0.2. All code is available at https://github.com/opensafely/comparative-ve-research and is shared openly for review and reuse under MIT open license. Code lists are available at https://www.opencodelists.org/. No person-level data are shared. Any reported figures based on counts below six are redacted or rounded for disclosure control.

### Patient and public involvement

We have developed a publicly available website https://opensafely.org/ through which we invite any patient or member of the public to contact us regarding this study or the broader OpenSAFELY project.

## Results

### Study population

A total of 361 287 health and social care workers aged 18-64 years receiving a first dose of BNT162b2 or ChAdOx1 vaccine between 4 January and 28 February 2021 and actively registered at a general practice using the TPP SystmOne clinical information system were identified, with 317 341 (87.8%) meeting the study eligibility criteria (supplementary tables S1 and S2 on bmj.com).

In total, 253 134 (79.8%) were vaccinated with BNT162b2, contributing 95 420 person-years of potential follow-up (including all person time before a censoring event, and possibly after an outcome event); and 64 207 (20.2%) were vaccinated with ChAdOx1, contributing 23 351 person-years.

**Fig1:**
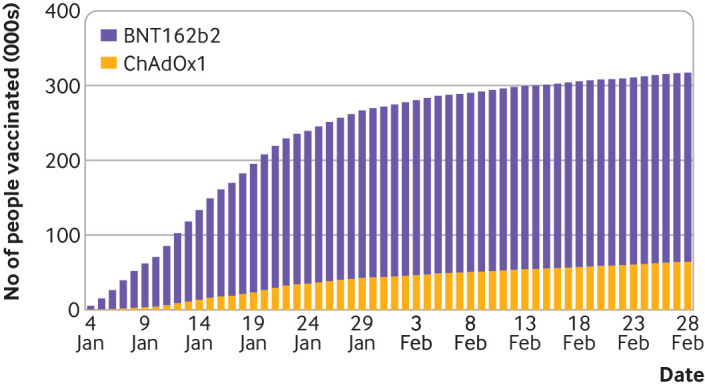
Cumulative enrolment of study participants over time, by covid-19 vaccine type (ChAdOx1 and BNT162b2)

Characteristics were largely well balanced between recipients of each vaccine, though regional and temporal differences in the distribution of each vaccine are notable (table 1). BNT162b2 was on average administered earlier than ChAdOx1 (fig 1; median day of vaccination 15 January for BNT162b2, 22 January for ChadOx1). BNT162b2 was relatively more likely to be administered in the South and East of England, and ChAdOx1 the Midlands and Northern England. Evidence of prior SARS-CoV-2 infection was higher in ChAdOx1 recipients (10.8% for BNT162b2, 14.1% for ChAdOx1), consistent with ChAdOx1 recipients being vaccinated later on average. The proportion of each clinical condition is slightly higher in ChAdOx1 recipients, though consistently under a 0.6% percent-point difference.

**Table 1:** Baseline characteristics of participants on day of covid-19 vaccination, by vaccine type. Values are numbers (percentages) of participants unless stated otherwise

Characteristic	BNT162b2 (n=253 134)	ChAdOx1 (n=64 207)
Age (years):		
18-30	41 086 (16)	10 464 (16)
30s	59 518 (24)	14 724 (23)
40s	64 553 (26)	16 286 (25)
50s	67 776 (27)	17 543 (27)
60-64	20 201 (8.0)	5,190 (8.1)
Sex:		
Female	200 149 (79)	49 331 (77)
Male	52 985 (21)	14 876 (23)
Ethnicity:		
White	211 463 (84)	54 287 (85)
Black	8 518 (3.4)	3 161 (4.9)
South Asian	23 140 (9.1)	4 701 (7.3)
Mixed	3 848 (1.5)	1 007 (1.6)
Other	6 165 (2.4)	1 051 (1.6)
Index of Multiple Deprivation (IMD):		
1 (most deprived)	36 850 (15)	10 300 (16)
2	47 279 (19)	12 116 (19)
3	55 832 (22)	13 751 (21)
4	57 499 (23)	14 353 (22)
5 (least deprived)	55 674 (22)	13 687 (21)
Region:		
North East and Yorkshire	53 522 (21)	16 418 (26)
East of England	62 377 (25)	11 861 (18)
Midlands	50 582 (20)	17 224 (27)
South West	35 948 (14)	5 056 (7.9)
London	10 405 (4.1)	2 148 (3.3)
North West	23 644 (9.3)	7 875 (12)
South East	16 656 (6.6)	3 625 (5.6)
Rural/urban category:		
Urban conurbation	61 699 (24)	19 295 (30)
Urban city or town	144 222 (57)	32 194 (50)
Rural town or village	47 213 (19)	12 718 (20)
Median (interquartile range) vaccination day (from 4 January 2021)	12 (7-18)	19 (13-33)
Body mass index >40 (kg/m^2^)	9 789 (3.9)	2 860 (4.5)
Chronic heart disease	9 207 (3.6)	2 527 (3.9)
Chronic kidney disease	1 994 (0.8)	551 (0.9)
Diabetes	12 674 (5.0)	3 339 (5.2)
Chronic liver disease	3 854 (1.5)	1 189 (1.9)
Chronic respiratory disease	2 579 (1.0)	731 (1.1)
Chronic neurological disease	6 063 (2.4)	1 620 (2.5)
Immunosuppressed	2 527 (1.0)	681 (1.1)
Asplenia or poor spleen function	1 704 (0.7)	481 (0.7)
Learning disabilities	187 (<0.1)	60 (<0.1)
Serious mental illness	1 276 (0.5)	434 (0.7)
Morbidity count:		
0	210 107 (83)	52 455 (82)
1	36 544 (14)	9 835 (15)
≥2	6 483 (2.6)	1 917 (3.0)
Prior SARS-CoV-2 infection	27 312 (11)	9 085 (14)
No of SARS-CoV-2 tests in previous 3 months:		
0	160 576 (63)	42 095 (66)
1-3	73 611 (29)	18 946 (30)
4-6	10 696 (4.2)	1 765 (2.7)
≥7	8 251 (3.3)	1 401 (2.2)

### Events

Over the duration of 118 771 person-years of follow-up there were 6962 positive SARS-CoV-2 tests, 282 covid-19 related A&E attendances, and 166 covid-19 related hospital admissions. While not a primary outcome, there were also 47 deaths from any cause, of which fewer than six were covid-19 related (supplementary table S3).

### Second doses

At 12 weeks (84 days), 95.4% of BNT162b2 recipients had received a second dose, compared with 90.8% of ChAdOx1 recipients. There were 413 (0.13%) participants who received a second dose within 20 weeks (140 days) that was not the same brand as the first dose, including Moderna mRNA-1273 vaccine second doses (supplementary fig S3, table S4).

### Comparative effectiveness

**Fig 2:**
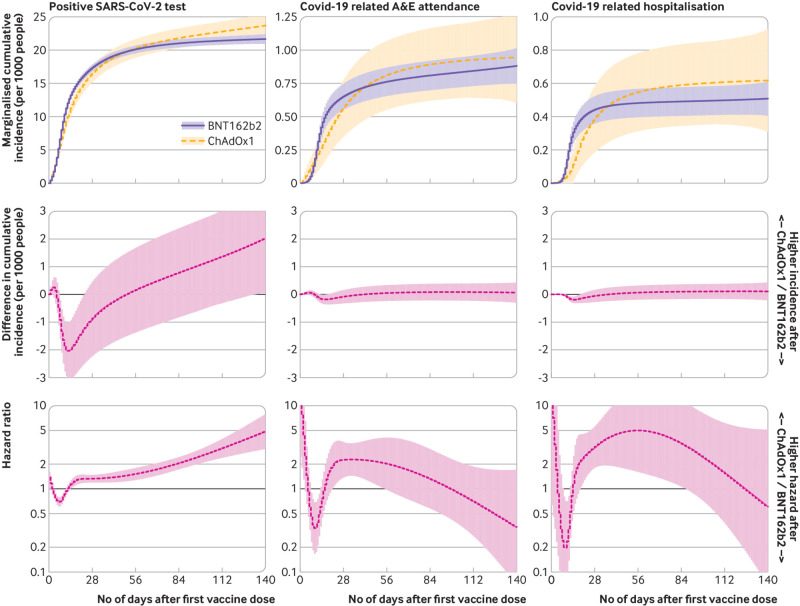
Comparative effectiveness of covid-19 vaccine ChAdOx1 and BNT162b2. For each outcome based on the fully adjusted model, the marginal cumulative incidence for ChAdOx1 and BNT162b2, their difference, and the hazard ratio are shown. The models with less extensive confounder adjustment gave similar estimates (supplementary fig S2), suggesting that recipients of each vaccine were similar after accounting for differences in vaccine allocation over space and time (as did all models). Models that assumed piecewise-constant hazards gave similar effect estimates (supplementary fig S3).

By six weeks (42 days) post-vaccination, before receipt of the second dose for the vast majority of participants, the ChAdOx1 versus BNT162b2 absolute risk difference per 1000 people (fig 2) for a positive SARS-CoV-2 test was −0.24 (95% confidence interval −1.71 to 1.22), for covid-19 related A&E attendances was 0.01 (−0.27 to 0.28), and for covid-19 related hospital admissions was 0.03 (−0.22 to 0.27).

At 20 weeks (140 days) post-vaccination, at which time most participants had received their second dose, the absolute risk difference per 1000 people for a positive SARS-CoV-2 test was 2.04 (0.04 to 4.04), for covid-19 A&E attendances was 0.06 (−0.31 to 0.43), and for covid-19 hospital admissions was 0.11 (−0.22 to 0.44). We note that, extrapolating beyond 20 weeks for positive SARS-CoV-2 tests, we would infer a small advantage for BNT162b2 in terms of absolute risk.

In general, events rates are much higher in the first few weeks after vaccination, and then there is a clear levelling-off after around 3-4 weeks for both vaccines, beyond which the event rates were very low. For covid-19 related hospital attendance and admission events in particular, the difference in the cumulative incidence (and confidence intervals) was well below one event per 1000 people in either direction. The estimated log hazard ratios were non-zero at certain periods (fig 2), but the underlying absolute risk was small enough that this did not manifest in substantial differences in the cumulative incidence beyond the fourth week.

A sensitivity analysis restricting comparative effectiveness estimates to those with no prior evidence of SARS-CoV-2 infection showed similar results (supplementary fig S5).

## Discussion

### Key findings

This observational study of 317 341 adult health and social care workers living in England found similar outcomes for those receiving the BNT162b2 or ChAdOx1 covid-19 vaccines during the first 20 weeks post-vaccination in the Alpha variant era. Consistent with the expected time to onset of vaccine induced immunity of around two weeks after vaccination, plus the delay from infection to the event of interest, we found that event rates tapered after the first few weeks. There were few covid-19 related hospital attendance and admission events after this period, suggesting strong protective effects for both vaccines, but limiting the power to reliably estimate comparative effectiveness with respect to severe covid-19 outcomes.

There is some evidence that, compared with ChAdOx1 recipients, there was better long term protection for BNT162b2 recipients, but they were also more likely to receive their second dose sooner, likely conferring a greater protective effect that is not accounted for in our intention-to-treat effectiveness estimates.

### Strengths and weaknesses

We used routinely collected health records with comprehensive coverage of primary care, hospital admissions, covid-19 testing, covid-19 vaccination, and death registrations to study vaccinated health and social care workers. This group were eligible for vaccination at the start of the UK’s vaccination programme because of their exposure to higher viral loads and the need to reduce enforced absences in essential healthcare workers during a global pandemic. As such, health and social care worker status is an important determinant of both vaccination time and SARS-CoV-2 infection, and a certain source of confounding in studies of vaccine effectiveness, which may be reduced by studying this group in isolation. Although it is not typically possible to identify health and social care workers in NHS primary care records, this information was comprehensively collected at the time of covid-19 vaccination and so is known for all vaccine recipients. Health and social care workers are the only early vaccinees who are relatively young and healthy, and were vaccinated during a period where infection rates were high and both vaccines were being widely administered.^3^ Overall, this provides a rare opportunity to study comparative effectiveness under conditions that, to some extent, approximate random vaccine allocation. However, some limitations remain.

Despite reasonable balance of vaccine allocation across baseline characteristics and adjustment for a range of potential confounders, the possibility of unmeasured confounding remains. The cold storage requirements of BNT162b2 meant that it was more likely to have been administered in acute NHS trusts and other large vaccination centres, which is a potential confounder due to, for instance, differences in viral exposure across these settings. Although we adjusted for region, rurality, and deprivation, we were unable to directly account for occupational differences that may affect both exposure risk and vaccine type. For instance, we had no means to quantify any differences in health and social care worker specialty, working hours, or patient contact time between the treatment groups, and only crude measures of health seeking behaviour via the number of prior SARS-CoV-2 tests. The primary care practices covered in this study are not geographically representative, as the TPP SystmOne software is not widely used in some regions such as London and the North West.

We were unable to fully investigate differences in protection against severe disease, in large part due to clear protective benefits of both vaccines, reducing the absolute numbers of events, and therefore statistical power, in the studied cohort.

Second dose effects were considered using an intention-to-treat approach, which does not account for potential differences in the timing of the second dose between vaccine types. However, we found that, by 12 weeks, more BNT162b2 recipients had received a second dose than ChAdOx1 recipients, which would bias effect estimates in favour of BNT162b2 assuming higher protection after a second dose of either vaccine. Regardless, there was insufficient power to reliably estimate comparative second dose effects, as event incidence at 12 weeks and beyond was low for both vaccines, with fewer than 10 positive tests per 1000-person-years and far fewer for covid-19 related A&E attendances and hospital admissions. The need for many thousands more person-years to detect differences in second dose effects is encouraging to the extent that it suggests extremely high protective effects for both vaccines, though declining background case rates in England over the duration of the study period (supplementary fig S4) would have also contributed to reduced event rates in both treatment groups.

### Findings in context

Several studies have estimated first dose effectiveness of covid-19 vaccines in observational data,^9^ including BNT162b2 and ChAdOx1 vaccines in similar populations^10^ ^11^ ^12^ ^13^ and in healthcare workers.^14^ ^15^ Vaccine protection is typically assessed by comparing vaccinated against unvaccinated person-time, or against the early post-vaccination period before anticipated onset of vaccine induced immunity. Such designs are vulnerable to confounding and selection bias—for example, vaccine prioritisation and eligibility policies and risk or health-seeking behaviours. In addition, differences in vaccine access and acceptance can cause substantial imbalance between vaccinated and unvaccinated groups that may be impossible to account for. Many of these biases can be bypassed when making direct comparisons between recipients of different vaccine types, if the relative performance of two vaccines is of interest.^16^ To our knowledge, the present study is the first to assess effectiveness of the BNT162b2 and ChAdOx1 vaccines in a head-to-head comparison in either an experimental or observational setting.

Our study suggests that there is little to differentiate the vaccines with respect to their protective effects against infection and hospital related outcomes from covid-19 within the first 20 weeks after the first dose in adult health and social care workers under 65 years old. This pushes decisions about which vaccine to favour onto other attributes such as safety profile, logistics, and cost. However, we cannot rule out larger differences in effectiveness in other settings. For instance, results seen in this cohort of predominantly healthy health and social care workers may not reflect comparative effectiveness in more vulnerable groups, such as elderly or immunosuppressed people; the dominant circulating variant during the period of study was the Alpha variant, but that has now been replaced by successor variants, against which vaccines may be less effective^12^; longer term effectiveness, and in particular the potential immunological waning, may differ.

### Conclusion

This study found no substantial differences in the incidence of SARS-CoV-2 infection or covid-19 related hospital events after vaccination with BNT162b2 or ChAdOx1 in a cohort of health and social care workers in England in the Alpha era. There were few severe covid-19 outcomes after the expected onset of vaccine induced immunity at around two weeks, reducing power to reliably compare effectiveness against these outcomes. Further studies are needed to assess comparative effectiveness against newer, more prevalent variants and to assess longer term effectiveness.

**What is already known on this topic**

- Phase III randomised controlled trials show clear evidence of efficacy of the BNT162b2 mRNA and the ChAdOx1 vaccines against covid-19
- Observational studies have provided further evidence of effectiveness in mass vaccine roll-outs
- No head-to-head comparison of these vaccines has been made in randomised trials, and such comparisons are difficult in observational data without administration of both vaccines across the same population at the same time

**What this study adds**

- By exploiting the concurrent roll-out of both vaccines in health and social care workers in England, we were able to make direct comparisons of first and second dose effectiveness between the vaccines
- We found similar incidence rates for infection and covid-19 related hospital attendance and admission

## Data Availability

We will share information and interpretation of our findings through press releases, social media channels, and plain language summary on the OpenSAFELY website (https://opensafely.org/research/).

## Web Extra

Extra material supplied by the author

## Notes

Contributors: BG, SJWE, JACS, EW, KB, CTR, AS, ACAG, and WJH conceived of the study; LS, BG, and RME acquired funding; WJH, EW, ACAG, KB, HIM, CTR, AS, JT, HJC, AJW, LAT, TP, EMFH, BMK, CEM, AM, LF, JACS, MAH, BG, IJD, and SWJE developed the methodology; WJH, ACAG, and EMFH conducted the formal analysis; LAT, CEM, AM, BMK, RC, HJC, AJW, WJH, EW, KB, CTR, AS, ACAG, RME, AYSW, RM, KW, HF, DJG, LS, and BG developed code lists; SB, DE, PI, GH, TW, SD, CB, JC, JP, FH, and SH were responsible for the software; WJH was responsible for visualisation; WJH, HJC, AJW, LF, EMFH, and ACAG were responsible for verification: WJH wrote the first draft, and all authors read and approved the final manuscript; AM, CB, LS, BG, and JM were responsible for information governance; JM, WJH, ACAG, and AM were responsible for project administration; BG is guarantor. The corresponding author attests that all listed authors meet authorship criteria and that no others meeting the criteria have been omitted.

The views expressed are those of the authors and not necessarily those of the NIHR, NHS England, Public Health England, or the Department of Health and Social Care.

For the purpose of Open Access, the author has applied a CC BY public copyright licence to any Author Accepted Manuscript (AAM) version arising from this submission.

Information governance and ethical approval: NHS England is the data controller; TPP is the data processor; and the key researchers on OpenSAFELY are acting with the approval of NHS England. This implementation of OpenSAFELY is hosted within the TPP environment which is accredited to the ISO 27001 information security standard and is NHS IG Toolkit compliant^17^; Patient data has been pseudonymised for analysis and linkage using industry standard cryptographic hashing techniques; all pseudonymised datasets transmitted for linkage onto OpenSAFELY are encrypted; access to the platform is via a virtual private network (VPN) connection, restricted to a small group of researchers; the researchers hold contracts with NHS England and only access the platform to initiate database queries and statistical models; all database activity is logged; only aggregate statistical outputs leave the platform environment following best practice for anonymisation of results such as statistical disclosure control for low cell counts.^18^ The OpenSAFELY research platform adheres to the obligations of the UK General Data Protection Regulation (GDPR) and the Data Protection Act 2018. In March 2020, the Secretary of State for Health and Social Care used powers under the UK Health Service (Control of Patient Information) Regulations 2002 (COPI) to require organisations to process confidential patient information for the purposes of protecting public health, providing healthcare services to the public and monitoring and managing the covid-19 outbreak and incidents of exposure; this sets aside the requirement for patient consent.^19^ Taken together, these provide the legal bases to link patient datasets on the OpenSAFELY platform. GP practices, from which the primary care data are obtained, are required to share relevant health information to support the public health response to the pandemic, and have been informed of the OpenSAFELY analytics platform. This study was approved by the Health Research Authority (REC reference 20/LO/0651) and by the LSHTM Ethics Board (reference 21863).

Transparency: The guarantor affirms that the manuscript is an honest, accurate, and transparent account of the study being reported; that no important aspects of the study have been omitted; and that any discrepancies from the study as planned have been explained.

Provenance and peer review: Not commissioned; externally peer reviewed.
