# Supplementary material for: Comparative effectiveness of ChAdOx1 versus BNT162b2 covid-19 vaccines in health and social care workers in England: cohort study using OpenSAFELY
Source: BMJ. 2022 Jul 20;378:e068946. doi: 10.1136/bmj-2021-068946 (PMC9295078; doi:10.1136/bmj-2021-068946)
Supplement: Supplementary file 1 — Appendix: Supplementary figures S1-S5, supplementary tables S1-S4 [file hulw068946.w1.pdf]

Comparative vaccine effectiveness of ChAdOx1 versus BNT162b2 in Health and Social Care workers in England: Supplementary materials

Figure S1a: Comparative effectiveness adjusting for region-specific calendar-time

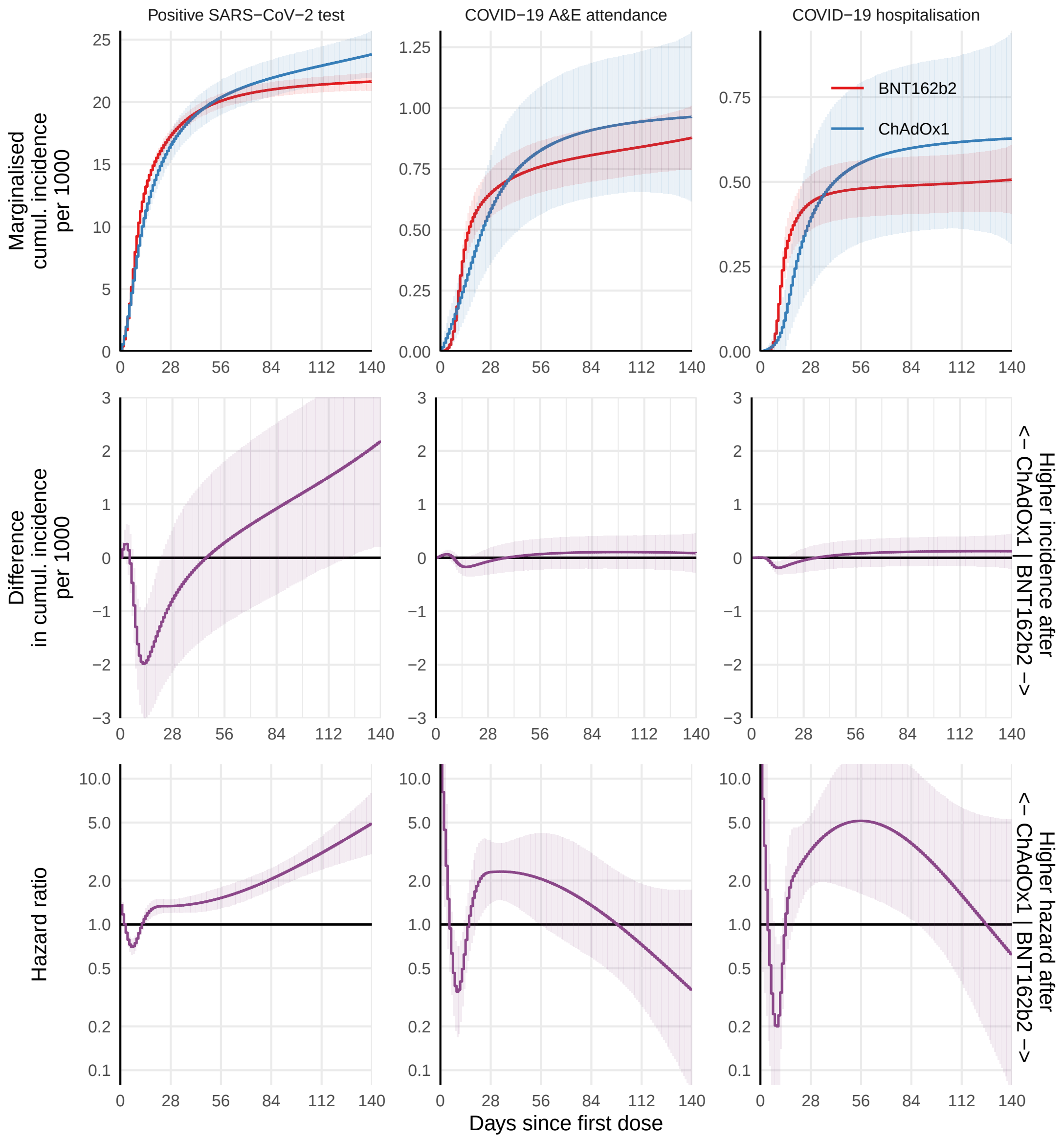

Figure S1b: Comparative effectiveness adjusting for region-specific calendar-time and demographic characteristics

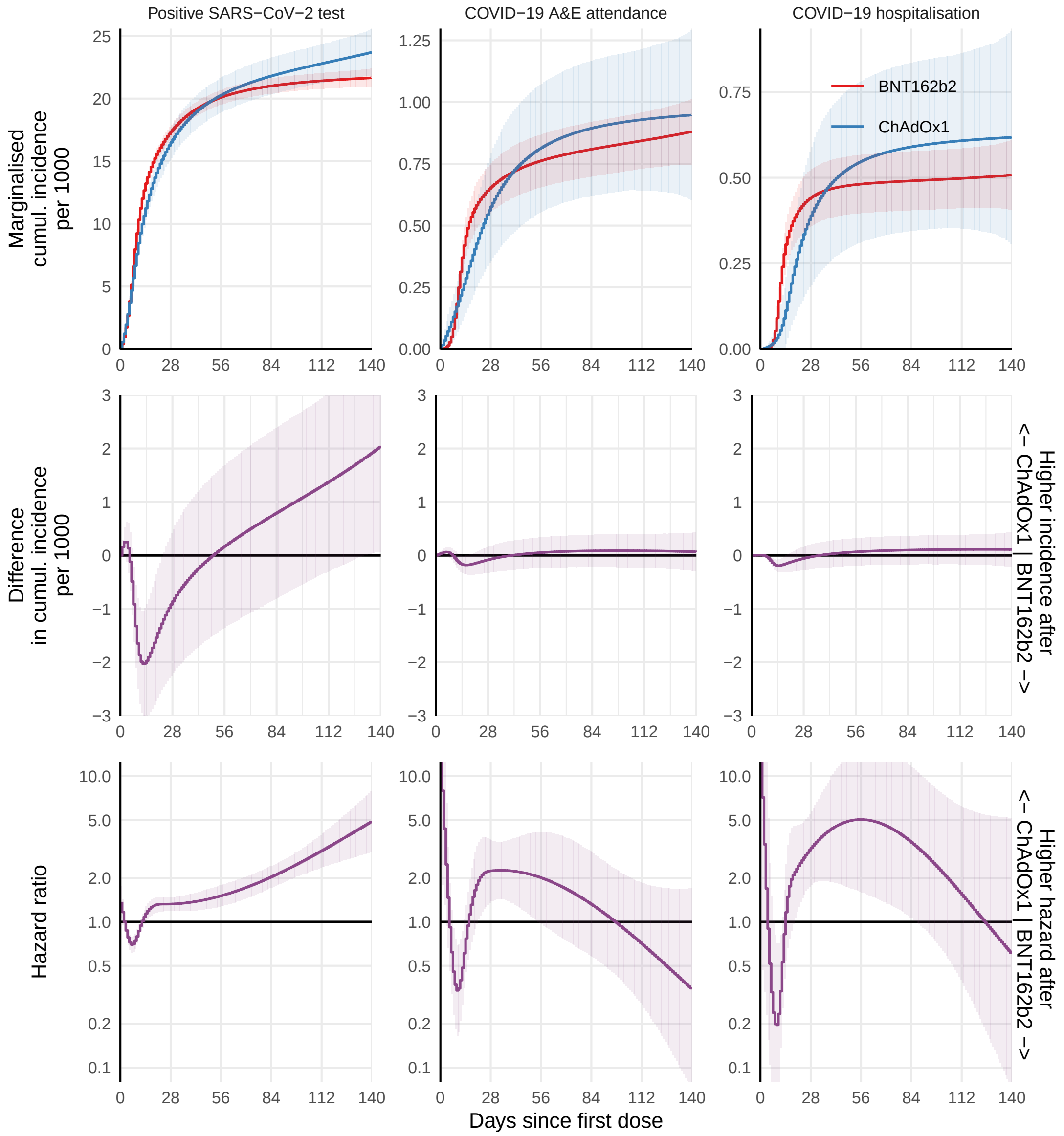

Figure S2: Fully adjusted comparative effectiveness using piecewise-constant estimates of the hazard

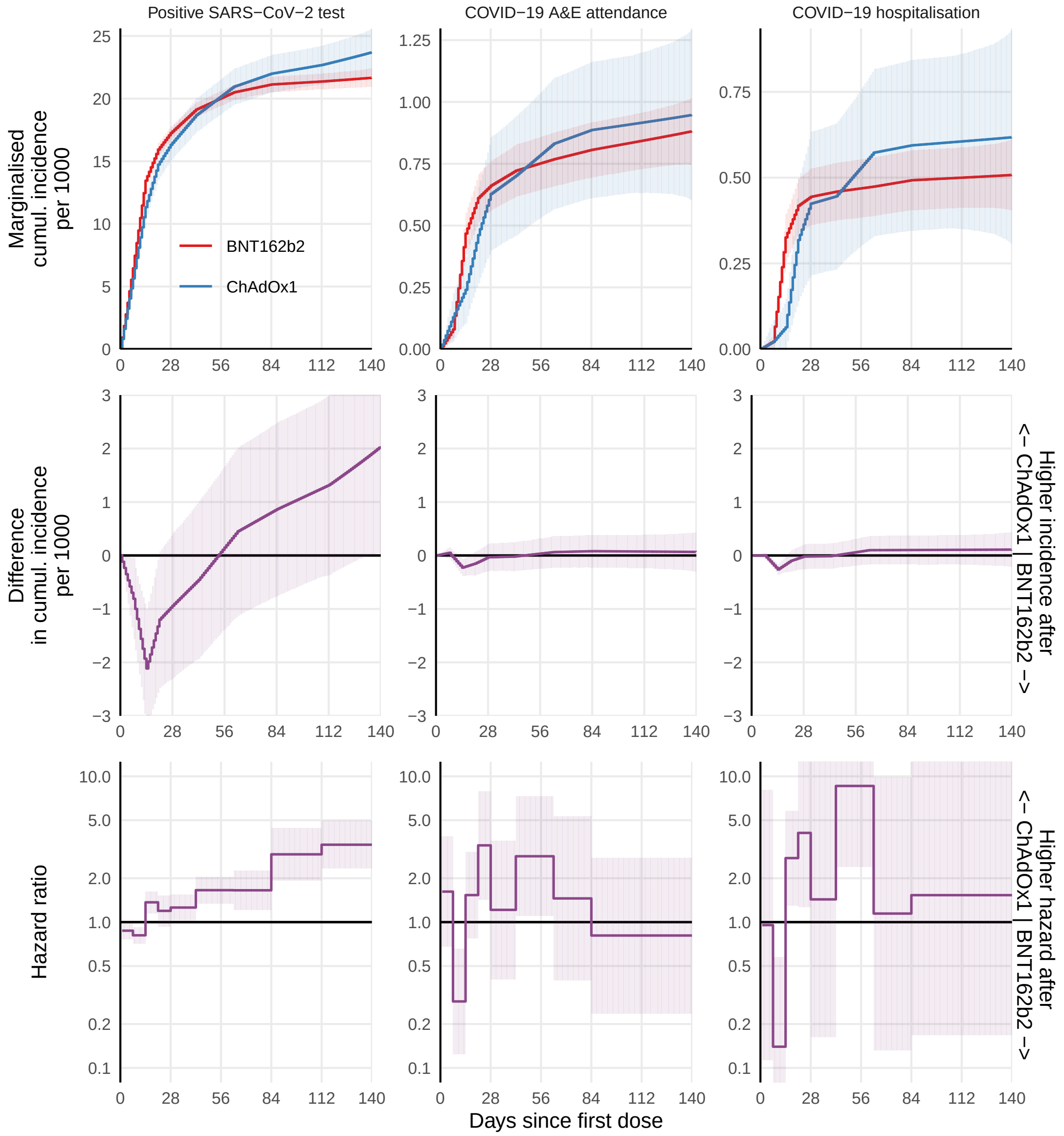

Figure S3: Second dose interval

Second dose events are censored by study end date, de-registration, death, and additionally by outcome-specific events.

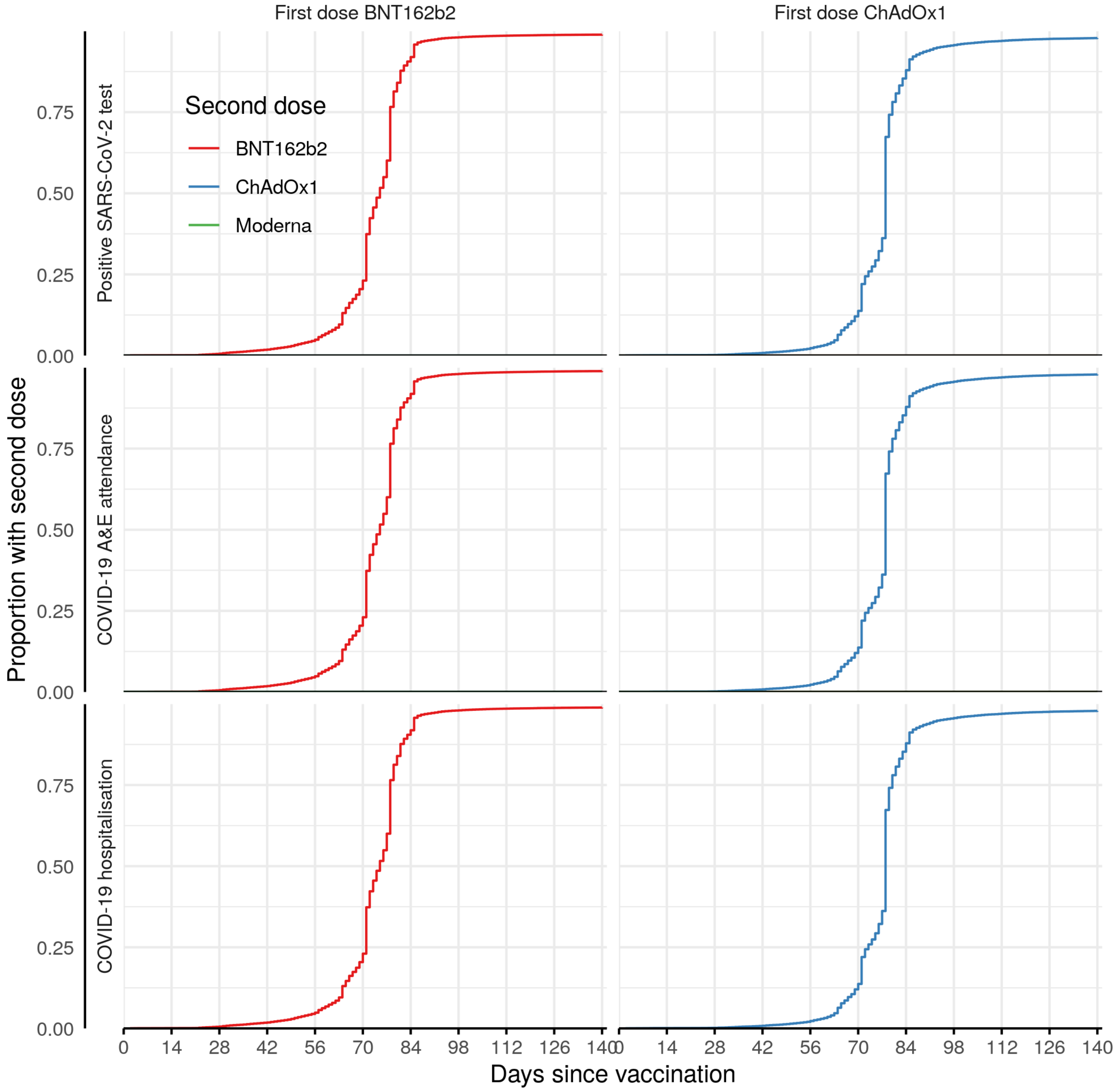

Figure S4: Event rates by calendar time

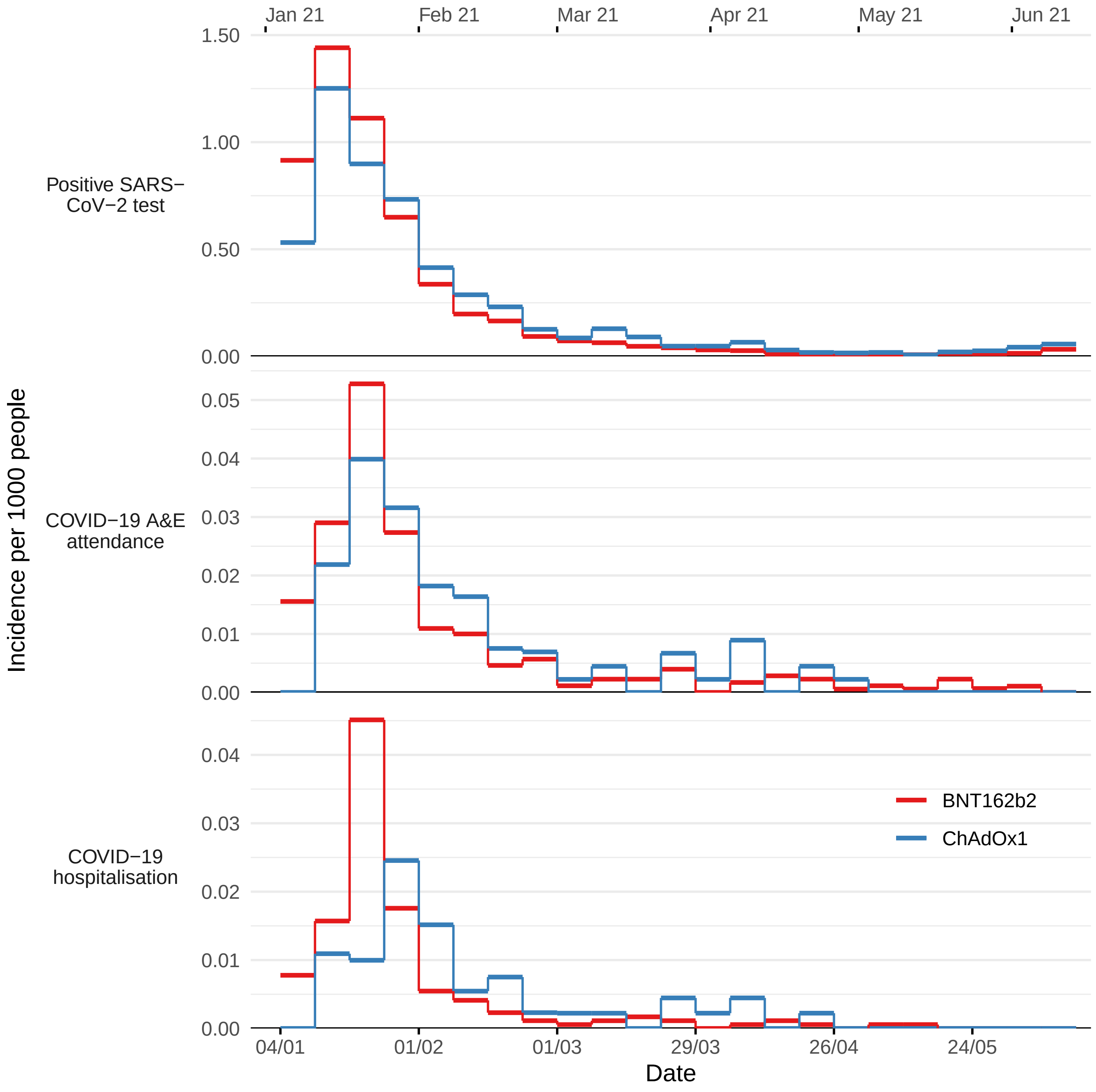

Figure S5: Fully adjusted comparative effectiveness in the subgroup of participants with no prior wvidence of SARS-CoV-2 infection

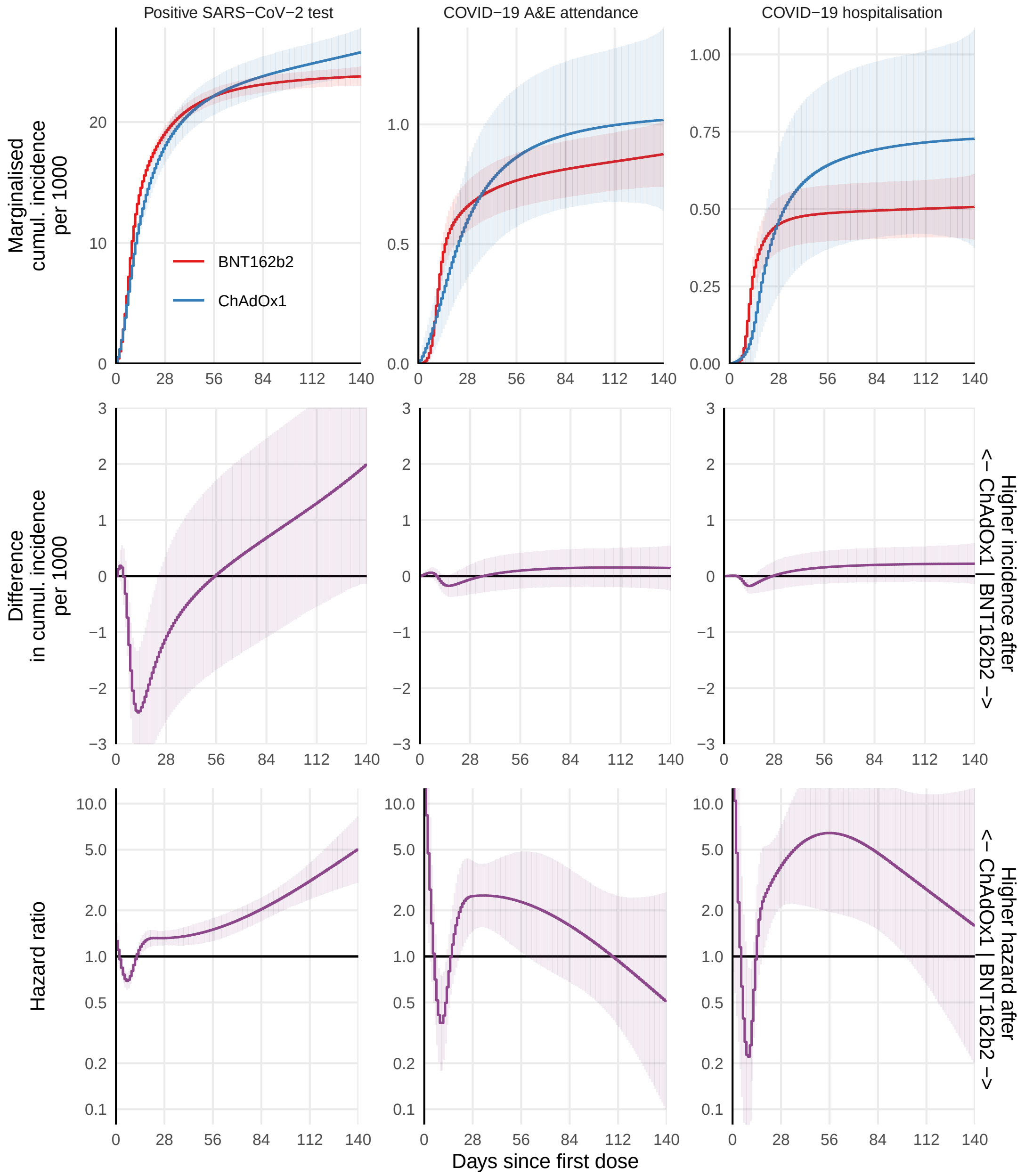

Table S1: Inclusion criteria

Participants	N	N excluded	% remaining
HCWs aged 18-64 receiving BNT162b2 or ChAdOx1 between 4 Jan and 28 Feb	361, 287	-	100.0%
with no missing demographic information	327, 254	34, 033	90.6%
who are not clinically extremely vulnerable	317, 341	9, 913	87.8%

Table S2: Baseline characteristics before eligibility exclusions

Characteristics for all HCWs aged 18-64 receiving a first dose of BNT162b2 or ChAdOx1 between 4 January and 28 February 2021 and actively registered at at TPP practice are reported, before exclusions due to missing demographic characteristics and Clinically Extremely Vulnerable status.

Characteristic	BNT162b2	ChAdOx1
Total N	286,559	74,728
Age		
18-30	47,700 (17%)	12,462 (17%)
30s	66,352 (23%)	16,769 (22%)
40s	72,266 (25%)	18,777 (25%)
50s	77,047 (27%)	20,481 (27%)
60-64	23,194 (8.1%)	6,239 (8.3%)
Sex		
Female	225,335 (79%)	57,290 (77%)
Male	[REDACTED]	[REDACTED]
Unknown	[REDACTED]	[REDACTED]
Ethnicity		
White	223,555 (83%)	58,810 (84%)
Black	9,381 (3.5%)	3,707 (5.3%)
South Asian	24,549 (9.2%)	5,188 (7.4%)
Mixed	4,123 (1.5%)	1,145 (1.6%)
Other	6,532 (2.4%)	1,147 (1.6%)
Unknown	18,419	4,731
IMD		
1 most deprived	40,175 (14%)	11,653 (16%)
2	51,761 (19%)	13,652 (19%)
3	61,325 (22%)	15,420 (21%)
4	63,102 (23%)	16,116 (22%)
5 least deprived	61,237 (22%)	15,362 (21%)
Unknown	8,959	2,525
Region		
North East and Yorkshire	60,707 (21%)	19,091 (26%)
East of England	70,597 (25%)	14,081 (19%)
Midlands	56,664 (20%)	19,752 (26%)
South West	41,161 (14%)	5,981 (8.0%)
London	11,672 (4.1%)	2,502 (3.3%)
North West	26,570 (9.3%)	9,030 (12%)
South East	19,088 (6.7%)	4,268 (5.7%)
Unknown	100	23
Rural/urban category		
Urban conurbation	67,119 (24%)	21,439 (30%)
Urban city or town	158,696 (57%)	36,582 (51%)
Rural town or village	51,879 (19%)	14,204 (20%)
Unknown	8,865	2,503
Vaccination day (from 4 January 2021)	12 (7, 19)	20 (13, 34)
Body Mass Index > 40 kg/m^2	11,102 (3.9%)	3,421 (4.6%)
Chronic heart disease	10,485 (3.7%)	3,055 (4.1%)
Chronic kidney disease	2,517 (0.9%)	806 (1.1%)
Diabetes	14,798 (5.2%)	4,490 (6.0%)
Chronic liver disease	4,426 (1.5%)	1,416 (1.9%)
Chronic respiratory disease	3,635 (1.3%)	1,266 (1.7%)

Chronic neurological disease	6,863 (2.4%)	1,941 (2.6%)
Immunosuppressed	4,465 (1.6%)	1,573 (2.1%)
Asplenia or poor spleen function	2,169 (0.8%)	654 (0.9%)
Learning disabilities	215 (<0.1%)	87 (0.1%)
Serious mental illness	1,397 (0.5%)	501 (0.7%)
Morbidity count		
0	235,874 (82%)	59,642 (80%)
1	42,344 (15%)	12,174 (16%)
2+	8,341 (2.9%)	2,912 (3.9%)
Prior SARS-CoV-2 infection	30,509 (11%)	10,486 (14%)
Number of SARS-CoV-2 tests in previous 3 months		
0	182,942 (64%)	49,148 (66%)
1-3	82,447 (29%)	21,857 (29%)
4-6	11,988 (4.2%)	2,099 (2.8%)
7+	9,182 (3.2%)	1,624 (2.2%)
Clinically Extremely Vulnerable	6,838 (2.4%)	3,578 (4.8%)

Table S3: Event rates

	BNT162b2		ChAdOx1	
Days since first dose	Events / person-years	Incidence	Events / person-years	Incidence
Positive SARS-CoV-2 test				
1-14	21 / 103	0.204	16 / 72	0.221
15-28	26 / 102	0.255	24 / 72	0.335
29-42	14 / 101	0.138	8 / 71	0.113
43-70	54 / 199	0.271	42 / 139	0.302
71-84	29 / 98	0.296	14 / 68	0.205
85-98	25 / 97	0.258	20 / 67	0.296
99-112	24 / 94	0.255	17 / 66	0.259
113-126	17 / 77	0.220	15 / 56	0.269
127-140	14 / 54	0.258	14 / 41	0.345
All	224 / 926	0.242	170 / 652	0.261
COVID-19 A&E attendance				
1-14	– / 103	–	– / 73	–
15-28	0 / 103	0	– / 72	–
29-42	– / 103	–	0 / 72	0
43-70	– / 206	–	– / 144	–
71-84	– / 103	–	0 / 72	0
85-98	0 / 102	0	0 / 72	0
99-112	0 / 100	0	– / 70	–
113-126	0 / 83	0	0 / 60	0
127-140	– / 59	–	0 / 44	0
All	7 / 964	0.007	8 / 679	0.012
COVID-19 hospitalisation				
1-14	7 / 103	0.068	– / 73	–
15-28	– / 103	–	6 / 72	0.083
29-42	6 / 103	0.058	– / 72	–
43-70	7 / 204	0.034	12 / 143	0.084
71-84	6 / 102	0.059	6 / 71	0.085
85-98	– / 102	–	– / 71	–
99-112	9 / 99	0.091	– / 69	–
113-126	– / 82	–	– / 59	–
127-140	6 / 58	0.103	– / 43	–
All	54 / 956	0.056	42 / 673	0.062
COVID-19 death				
1-14	– / 103	–	– / 73	–
15-28	– / 103	–	0 / 73	0
29-42	0 / 103	0	0 / 72	0
43-70	0 / 206	0	– / 144	–
71-84	0 / 103	0	0 / 72	0
85-98	0 / 103	0	0 / 72	0
99-112	0 / 101	0	0 / 71	0
113-126	0 / 84	0	0 / 60	0
127-140	0 / 59	0	– / 44	–
All	– / 965	–	– / 681	–
Second dose				

1-14	0 / 103	0	0 / 73	0
15-28	0 / 103	0	0 / 73	0
29-42	0 / 103	0	0 / 72	0
43-70	0 / 206	0	0 / 144	0
71-84	109 / 102	1.066	70 / 72	0.978
85-98	283 / 93	3.029	217 / 65	3.323
99-112	293 / 80	3.642	178 / 57	3.146
113-126	127 / 59	2.145	78 / 43	1.796
127-140	0 / 41	0	0 / 31	0
All	812 / 893	0.910	543 / 630	0.862

Table S4: Vaccine type for second doses

First dose	Matching second dose	N	%
BNT162b2	no	288	0.1%
BNT162b2	yes	249,091	98.4%
BNT162b2	no second dose at 20 weeks	3,755	1.5%
ChAdOx1	no	125	0.2%
ChAdOx1	yes	62,463	97.3%
ChAdOx1	no second dose at 20 weeks	1,619	2.5%
